# Valvular and perivalvular thrombosis following self-expandable aortic valve replacement: analysis of 100 multi-detector computed tomography scans

**DOI:** 10.1093/ehjopen/oeae085

**Published:** 2024-10-15

**Authors:** Marco Moscarelli, Gregorio Zaccone, Adriana Zlahoda-Huzior, Vincenzo Pernice, Sabrina Milo, Francesco Violante, Francesca Trizzino, Dariusz Dudek, Giuseppe Speziale, Patrizio Lancellotti, Khalil Fattouch

**Affiliations:** Department of Cardiovascular Surgery, Maria Eleonora Hospital, Viale della Regione Siciliana Nord Ovest, 1571, 90135 Palermo PA, Italy; Department of Surgery & Cancer, Faculty of Medicine, Hammersmith Hospital, 72 Du Cane Rd, London W12 0HS, UK; Department of Cardiovascular Surgery, Maria Eleonora Hospital, Viale della Regione Siciliana Nord Ovest, 1571, 90135 Palermo PA, Italy; Department of Measurement and Electronics AGH University of Krakow, Poland, al. A. Mickiewicza 30 / B1 30-059 Kraków; SimHub, VIRMED Sp. z o. o.Ul. Miechowska 5B / 1, 30-055 Kraków, Polska NIP 6772492319; Department of Cardiovascular Surgery, Maria Eleonora Hospital, Viale della Regione Siciliana Nord Ovest, 1571, 90135 Palermo PA, Italy; Department of Radiology, Maria Eleonora Hospital, Viale della Regione Siciliana Nord Ovest, 1571, 90135 Palermo PA, Italy; Department of Radiology, Maria Eleonora Hospital, Viale della Regione Siciliana Nord Ovest, 1571, 90135 Palermo PA, Italy; Department of Cardiovascular Surgery, Maria Eleonora Hospital, Viale della Regione Siciliana Nord Ovest, 1571, 90135 Palermo PA, Italy; Jagiellonian University Medical College, Świętej Anny 12, 31-008 Kraków, Poland; Department of Cardiovascular Surgery, Anthea Hospital, GVM Care & Research, Via Camillo Rosalba, 35/37, 70124 Bari BA, Italy; Département des sciences cliniques, Cardiologie-Pathologies spéciales et réhabilitation, GIGA Institute, B36 Quartier Hôpital 4000 Liège, Belgique; Kore University, Faculty of Medicine, Piazza dell'Università, 94100 Enna EN, Italy

**Keywords:** Transcatheter aortic valve replacement, Subclinical leaflet thrombosis, Multi-detector computed tomography, Hypoattenuated lesion

## Abstract

**Aims:**

Subclinical thrombosis may represent an early stage of prosthesis structural disease. Most of the available evidence on the incidence, location, predictors, and consequences of thrombosis comes from studies that have employed balloon-expandable valves. We aimed to describe the different localisations of valvular and perivalvular thrombosis and analyse prosthesis-host multi-detector computed tomography predictors in the context of self-expandable prosthesis. Additionally, we aimed to assess the impact of valvular and perivalvular thrombosis on prosthesis performance and subsequent clinical outcomes.

**Methods and results:**

This analysis includes 100 consecutive patients with normal renal function who underwent transcatheter aortic valve replacement using Evolut R and received multi-detector computed tomography and transthoracic bi-dimensional echocardiography at the 6 month follow-up. Leaflet thrombosis was detected in 18 (18%) patients; 6 (6%) had at least one leaflet with severe thrombosis. Thrombosis of the anatomic sinus was detected in 24 patients (24%) and was more prevalent in the non-coronary sinus. Subvalvular thrombosis with partial or complete circumferential involvement of the prosthesis inner skirt was diagnosed in 23 patients (23%). Bicuspid valve was the predictor with highest association with hypoattenuated lesions [least absolute shrinkage and selection operator coefficient 0.35, 95%, confidence interval (CI) 0.21–0.68]. There was no difference in terms of haemodynamic structural valve dysfunction, neurological events, and re-hospitalisation between the groups with and without thrombosis (hazard ratio: 0.86, 95% CI: 0.24–3.06, *P* = 0.82).

**Conclusion:**

This study showed that in a relatively low-risk population, valvular and perivalvular thrombosis were not rare phenomena following transcatheter aortic valve replacement at early follow-up. Bicuspid valve showed the strongest association with post-implant thrombosis.

## Graphical abstract

**Graphical Abstract:**
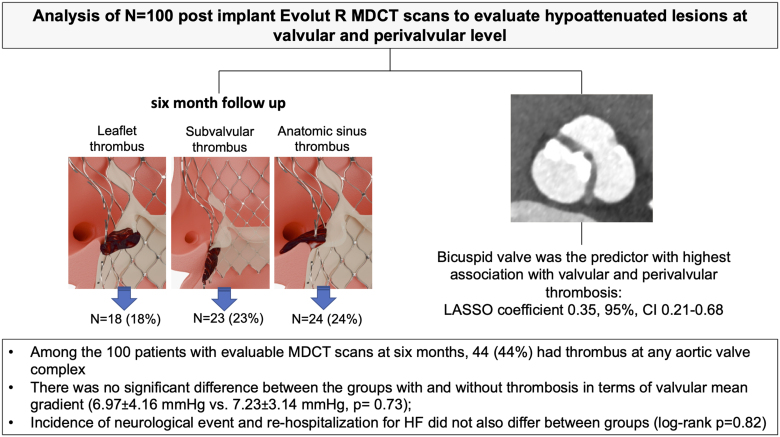

## Introduction

Transcatheter aortic valve replacement (TAVR) is currently a therapeutic option for low-risk populations.^1,2^ Owing to the prolonged life expectancy of these patients, assessment of the signs and predictors of early prosthesis dysfunction is needed.

While transcatheter aortic valves exhibit a relatively low incidence of structural valve deterioration and non-structural valve dysfunction,^3^ recent multi-detector computed tomography (MDCT) studies^4–6^ have shown a worrisome incidence of hypoattenuated lesions that suggest the presence of subclinical thrombus even at early follow-up. For example, Choi and colleagues reported an incidence of valve thrombus as high as 14.2%.^4^

**Figure 1:**
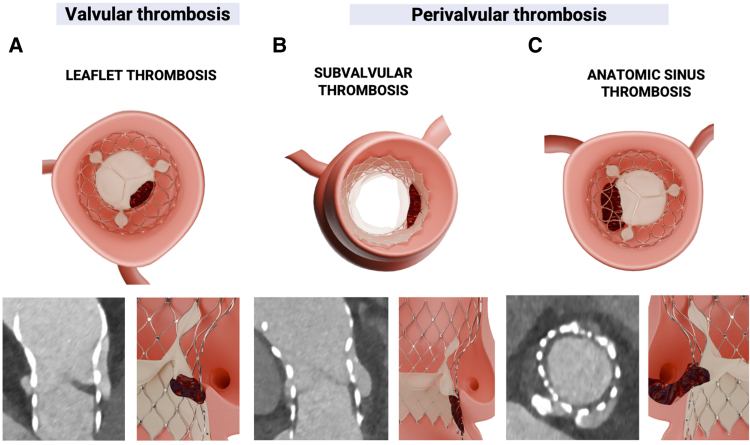
Schematic and multi-detector computed tomography representation of different locations of hypoattenuated lesions that suggest the presence of thrombus at: (*A*) valvular level; (*B*) subvalvular level (prosthesis inner skirt); (*C*) anatomic sinus level.

Notably, it has been demonstrated that the thrombus may affect not only the prosthetic leaflets but also the perivalvular components, such as the sinus of Valsalva and prosthetic inner skirt (*Figure 1A–C*). Whether these lesions represent an early stage of structural valve disease or a reversible phenomenon remains under investigation.^7^ Mechanisms that cause thrombus are multifactorial, and include laminar blood flow disruption following TAVR and the persistence of the native calcified leaflet at the level of the anatomic sinus that ultimately may act as ‘stagnation area’.^7^

The evaluation of thrombosis of the aortic valve complex (inclusive of valvular and perivalvular) and the examination of ‘device–host’ predictors of thrombosis have primarily been confined to balloon-expandable prostheses, with insufficient data available pertaining to self-expandable prostheses.^8^

Our preliminary report is the only study to have specifically investigated valvular and perivalvular thrombosis in the context of self-expandable prostheses.^6^ The report demonstrated that the incidence of hypoattenuated lesions at the leaflet, sinus, and subvalvular levels was as high as 16, 18, and 16%, respectively, and was more frequent in patients with native bicuspid valves.^6^ However, the findings were undermined by the ambidirectional nature of the study and the limited sample size.

We are now presenting the longitudinal follow-up of a larger cohort of patients with the following aims:

- To describe the frequency and different localisations of valvular and perivalvular thrombosis in the context of self-expandable prostheses (Evolut R, Medtronic Inc., Minneapolis, MN, USA).
- To evaluate ‘prosthesis—host’ predictors of aortic valve complex thrombosis, including (i) prosthesis eccentricity measured at six predefined levels, (ii) commissural alignment, (iii) leaflet expansion, (iv) implantation depth, (v) valve-to-coronary and non-coronary sinus distance, and (vi) native bicuspid valve.
- To assess prosthesis haemodynamic performance and clinical outcomes.^9^

## Methods

### Study design and participant

The present longitudinal study was the MDCT substudy of the ‘EndoTAVI Project’, a European grant funded study (PO-FESR 2014–2020) to evaluate predictors of early dysfunction after surgical and TAVR (www.endotavi.it), conducted at Maria Eleonora Hospital GVM Care & Research, a tertiary university centre in Palermo, Italy.

**Figure 2:**
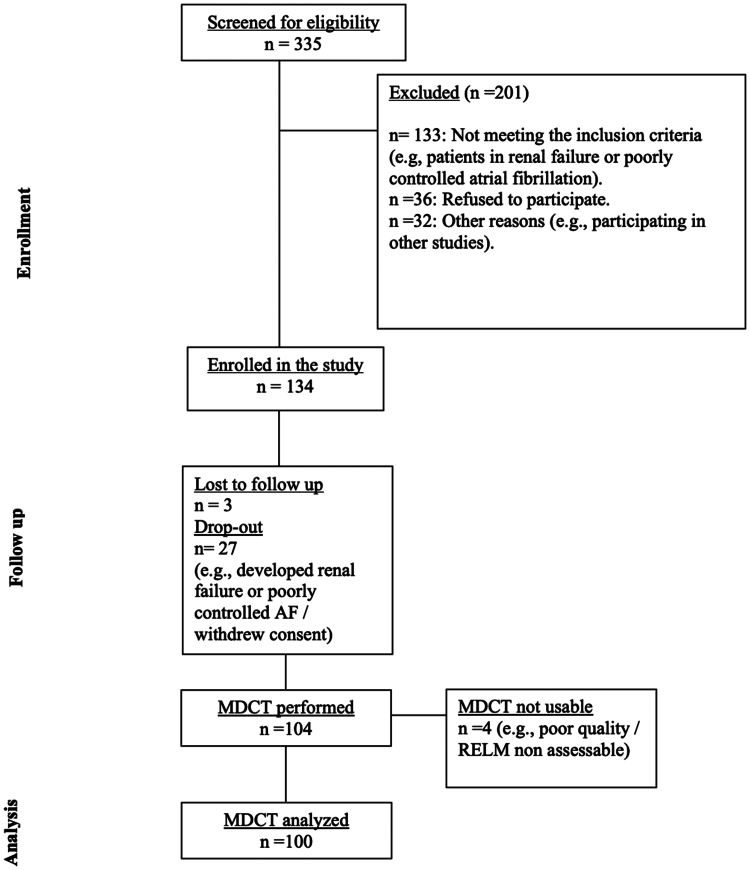
Study flow.

The study included 100 consecutive patients with severe aortic valve stenosis who underwent trans-femoral TAVR with Evolut R between January 2019 and September 2023, and 2D transthoracic echocardiography (TTE) and MDCT evaluation at 6 month follow-up (*Figure 2*: Study flow).

The full inclusion and exclusion criteria have been reported elsewhere^6^ and are provided in the Supplementary material.

In brief, the key exclusion criteria were moderate–severe renal insufficiency prohibiting MDCT imaging (estimated glomerular filtration rate < 40 mL/min/1.73 m^2^ of body surface area) or atrial fibrillation that could not be controlled to a ventricular response rate < 60 beats/min. New/direct oral anticoagulants, vitamin K antagonist or dual antiplatelet therapy did not represent an exclusion criterion ‘*per se*’. Patients were discharged on lifelong aspirin unless oral anticoagulants or dual antiplatelet therapy were otherwise indicated.

The study was performed in accordance with the Second Declaration of Helsinki, followed by the Strengthening the Reporting of Observational Studies in Epidemiology recommendations,^10^ and was approved by the Scientific Ethics Committee of the University of Palermo [Comitato Etico (Ce) University of Palermo, latest protocol approval 1/2020]. All patients provided written informed consent before enrolment in the study.

### Outcomes of interest

The primary outcome was:

- MDCT adjudication of hypoattenuated lesions at the valvular and perivalvular levels as reported below.

The secondary outcomes were as follows:

- Assessment of the predictors of any hypoattenuated lesions (i.e. leaflet, anatomic sinus, and subvalvular level);
- Evaluation of bioprosthetic valve dysfunction according to the Valve Academic Research Consortium-3 criteria (VARC-3) haemodynamic definition (HSVD), that included increase in mean transvalvular gradient ≥ 10 mmHg resulting in mean gradient ≥ 20 mmHg with concomitant decrease in effective orifice area (EOA) ≥ 0.3 cm^2^ compared with echocardiographic assessment performed at hospital discharge.^9^
- Evaluation of early clinical outcomes such as transient ischaemic attack (TIA), stroke, and cardiovascular re-hospitalization for procedural—valvular and non-valvular related causes.^9^

Echocardiographic data were obtained at baseline, hospital discharge, and 6 month follow-up. As secondary echocardiographic outcome, non-structural valve dysfunction, defined as moderate total aortic regurgitation or moderate/severe prosthesis–patient mismatch, was also assessed.^9^

### Device–host interaction and valvular and perivalvular thrombus multi-detector computed tomography imaging analysis

Pre- and post-procedural contrast—enhanced MDCT scans were performed using Siemens Healthcare GmbH SOMATOM Drive (VB 20, 2019). The analysis was performed using dedicated post-processing software (Syngo.via™ Siemens Healthcare GmbH, Forchheim, Germany).

### Hypoattenuated leaflet lesions

It was defined as hypoattenuated leaflet thickening (HALT) [< 200 Hounsfield units (HU)] of meniscal shape (> 2 mm) on the prosthetic leaflet surface (one or more leaflets) visualized during the diastolic phase of leaflet coaptation on at least two planes^11^ (*Figure 1A*). This condition is also known as subclinical leaflet thrombosis (SLT).^11^ The extent of HALT was graded on the long axis regarding involvement along the curvilinear leaflet beginning at the base using a 5-tier grading scale: no HALT, HALT ≤ 25%, HALT > 25 to 50%, HALT > 50 to 75%, and HALT > 75%.^12^ Per-patient severity was defined as the leaflet with the highest grade of HALT.^12^ Restricted leaflet movement (RELM) was defined as a hypoattenuated lesion visualised during systolic phase-maximal leaflet opening, which determines restricted mobility in one or more leaflets. RELM was further categorised as no RELM, RELM ≤ 25%, RELM > 25–50%, RELM > 50% −75%, and RELM > 75%.^12^ HALT with RELM > 50% signified an empirically set threshold for significantly reduced leaflet motion.^11^

### Hypoattenuated subvalvular lesion

It was defined as circumferential hypoattenuating low-density thickening (< 200 HU) of the prosthetic pericardial inner skirt ≥ 2 mm in diameter, visualised on at least two planes, extending from the nadir of the prosthetic leaflets towards the prosthesis inflow (*Figure 1B*).^5,6^

### Hypoattenuated anatomic sinus lesion

It was defined as hypoattenuated thickening (< 200 HU), at least > 2 mm in diameter, within the anatomic sinus (*Figure 1C*), without enhancement of the contrast media between the sinus of Valsalva and implanted prosthesis.^6,12^ Lesions on the left, right, and non-coronary cusps were assessed.

### Device–host characteristics

The following device–host characteristics were evaluated.

#### Prosthesis eccentricity

**Figure 3:**
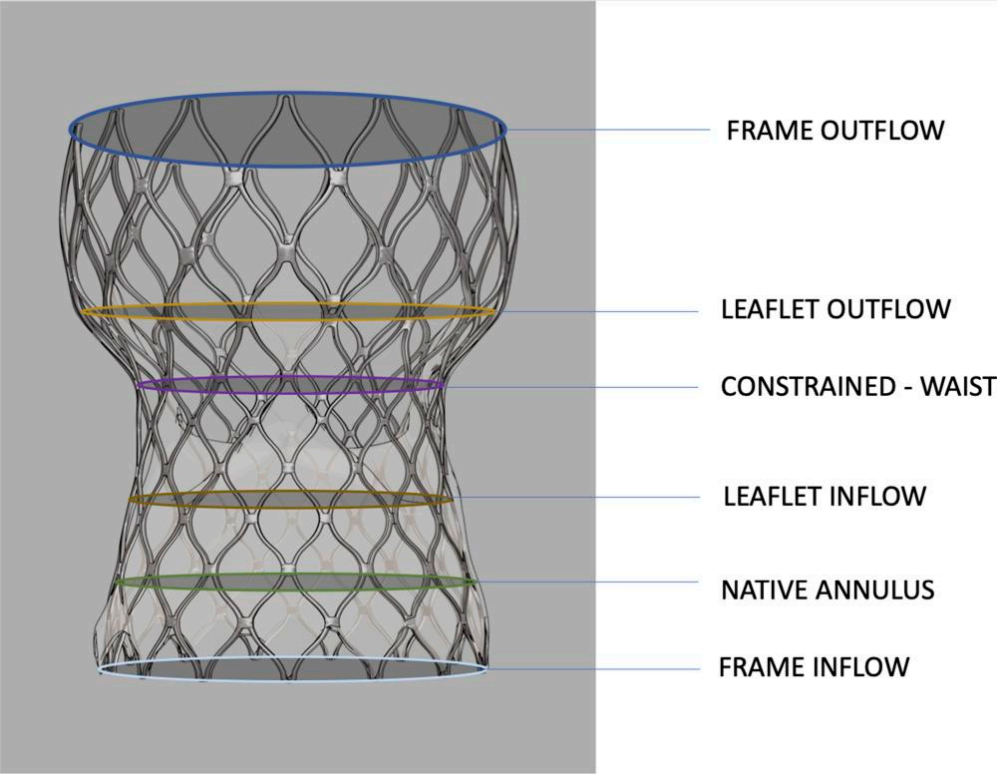
Multi-detector computed tomography measurements of major and minor diameters were performed at six levels of the prosthetic valve (Evolut R, medtronic): (i) frame inflow (nadir of the prosthesis); (ii) native annulus; (iii) leaflet inflow (nadir of the prosthetic leaflets); (iv) prosthesis waist (the waist between the leaflet outflow and the leaflet inflow levels); (v) leaflet outflow (the three commissural tabs of the prosthetic leaflets); (vi) frame outflow (tip of the prosthesis).

Given the Evolut R hourglass shape with varying cross-sectional areas throughout the frame, the minimum (*D*_min_) and maximum (*D*_max_) prosthesis diameters were measured at six prespecified levels, as shown in *Figure 3*, which included: (i) frame inflow (nadir of the prosthesis), (ii) native annulus, (iii) leaflet inflow (nadir of the prosthetic leaflets), (iv) prosthesis waist between the leaflet outflow and the leaflet inflow levels, (v) leaflet outflow (three commissural tabs of prosthetic leaflets), and (iv) frame outflow (tips of prosthesis), as previously described.^6,8^

The prosthesis eccentricity was calculated at each of the above-described levels as follows:

$$\mathrm{Eccentricity}\;\mathrm{index}=\sqrt{1-\frac{{\left(D_{\min}\right)}^{2}}{{\left(D_{\max}\right)}^{2}}}.$$

The eccentricity index ranges from 0 to 1. A larger eccentricity index (close to 1) represents a configuration similar to that of an oval shape, whereas a smaller index (close to 0) represents a circular orientation.^8^

#### Commissural alignment

Commissural alignment was defined by the Alignment of Transcatheter Aortic-Valve Neo-Commissures consortium according to the commissural offset, and an angle ≥30° signified an empirically set threshold for significant commissural misalignment^13^ (see Supplementary material online, *Figure S1*).

#### Leaflet expansion

Leaflet expansion was calculated by evaluating the angle formed by the border stent struts relative to each leaflet and prosthesis centre point, represented by the coaptation level. Full leaflet expansion was assumed to be 120° and asymmetric leaflet expansion was calculated as the sum of the difference between 120° and each measured leaflet angle^14^ (see Supplementary material online, *Figure S2*).

#### Implantation depth

Since there is no specific recommendation in the VARC-3 criteria,^9^ we measured the implantation depth by calculating, from the pre- and post-implant MDCT, the distance from the sinotubular junction and the virtual basal ring at the nadir of the left, right and non-coronary leaflet and the distance from the sinotubular junction and the prosthesis frame inflow at the same nadir, as shown in Supplementary material online, *Figure S3A*–*G*.

#### Valve-to-coronary and non-coronary sinus distance

It was defined as the greatest distance measured from the prosthesis cage to the edge of the right and left coronary sinuses, respectively and the non-coronary sinus^15^ (see Supplementary material online, *Figure S4A*), and was considered the bi-dimensional surrogate measure of the ‘anatomic sinus’, defined as the residual space between the prosthesis cage and the aortic wall (see Supplementary material online, *Figure S4B*).

#### Morphology and classification of bicuspid valve

We used the Sievers and Schmidtke bicuspid morphological classification based on the number of raphes (Type 0, I, and II) since it has been the most widely adopted^16^ (see Supplementary material).

### Lesion adjudication and grading

MDCT hypoattenuated lesion adjudication and grading were performed by two experienced radiologists (G.S. and F.V.) and one biomedical engineer (A.Z.-H.). The device–host characteristics (i.e. prosthesis eccentricity, implantation depth, etc.) were analysed by M.M. and G.Z. Any disagreements were resolved by the last author.

The first, second, and last authors, and F.T. (study co-ordinator) had full access to the data. The authors vouch for the accuracy and completeness of the data and fidelity to the protocol.

### Statistical analysis

Data were prospectively collected in an *ad hoc* digital case report form. The distribution of data was checked for normality before further analysis using the Shapiro–Wilk test. Continuous data were presented as mean ± Sd. The unpaired *t*-test or Wilcoxon rank-sum test was used for statistical comparison. Categorical data are presented as proportions and were compared using the χ^2^ test.

To mitigate overfitting and the effect of multicollinearity, we conducted least absolute shrinkage and selection operator (LASSO) regression to identify potential MDCT factors related to hypoattenuated lesions at any valve complex. The best lambda was determined with tenfold cross validation. The magnitude of non-zero beta coefficients indicates the strength of the relationship between the predictor and the outcome. Larger coefficients suggest a stronger effect on the outcome, while smaller coefficients suggest a weaker effect. Residual bootstrap procedure was applied to produce 95% confidence interval (CI) for each regression coefficient. A time-to-event analysis using Kaplan–Meier estimates was used for analyses of neurological events or re-hospitalisation for heart failure; the log-rank test and hazard ratio (HR) were used for outcome comparisons between exposure (presence/absence of thrombus).

Hypothesis testing for equivalence was set at a two-tailed level of 0.05. Analyses and data modelling were performed using R-project (version 2023.12. R-project for Statistical Computing) and R-studio (www.rstudio.com).

## Results

### Patient population, baseline, and procedural characteristics

The study flow/consort diagram is shown in *Figure 2*. Among the 335 patients screened for study inclusion, a total of 100 had available MDCT scans with sufficient image quality to allow the assessment of valvular and perivalvular thrombosis and leaflet excursion (i.e. evaluation of RELM). The overall patient populations and baseline characteristics are shown in *Table 1*.

**Table 1:** Characteristics of the patients at baseline and procedural details according to thrombus at any valve complex

	Overall cohort	Valvular and/or perivalvular thrombosis^a^		*P*-value
	*n* = 100			
		No	Yes	
		*n* = 56	*n* = 44	
Age, years	78.2 (5.8)	78.2 (5.9)	78.3 (5.9)	0.94
BSA^b^, m^2^	1.7	1.8 (0.2)	1.7 (0.2)	0.22
BMI^c^	27.8 (5.0)	28.25 (5.1)	27.43 (4.9)	0.42
BMI≥30	26 (26)	14 (25)	12 (27.3)	0.97
Male sex, no. (%)	53 (53.0)	33 (58.9)	20 (45.5)	0.25
Euroscore II, %	2.03 (1.02)	1.92 (0.7)	2.1 (1.2)	0.22
Low flow—low gradient, *n* (%)	11 (11.0)	8 (14.3)	3 (6.8)	0.38
NYHA functional Class III–IV, no. (%)	13 (13.0)	9 (16.1)	4 (9.1)	0.46
NIDDM/IDDM, no. (%)	30 (30.0)	17 (30.4)	13 (29.5)	0.99
Hypertension, no. (%)	98 (98.0)	55 (98.2)	43 (97.7)	0.99
COPD, no. (%)	18 (18.0)	11 (19.6)	7 (15.9)	0.82
Previous stroke/TIA, no. (%)	4 (4.0)	2 (3.6)	2 (4.5)	0.99
Previous PCI, no. (%)	22 (22.0)	12 (21.4)	10 (22.7)	0.99
Previous cardiac surgery, no. (%)	9 (9.0)	3 (5.4)	6 (13.6)	0.27
Previous MI, no. (%)	17 (17.0)	8 (14.3)	9 (20.5)	0.58
Coronary artery disease, no. (%)	28 (28.0)	15 (26.8)	13 (29.5)	0.93
Serum creatinine, mg/dL	0.8 (0.2)	0.8 (0.2)	0.8 (0.2)	0.46
Creatinine clearance by Cockcroft–Gault formula (mL/min)	71.4 (28.1)	77.5 (32.2)	66.7 (19.5)	0.14
Pre-existing pacemaker or defibrillator, no. (%)	11 (11.0)	7 (12.5)	4 (9.1)	0.82
History of right bundle branch block, no. (%)	3 (3.0)	2 (3.6)	1 (2.3)	0.99
Treatment with vitamin K antagonist, no. (%)	2 (2.0)	1 (1.8)	1 (2.3)	0.99
Treatment with direct oral anticoagulant, no. (%)	5 (5.0)	4 (7.1)	1 (2.3)	0.51
Dual antiplatelets therapy, no. (%)	23 (23.0)	15 (26.8)	8 (18.2)	0.43
*Procedural characteristics:*				
Valve size	–	–	–	0.01
Evolut R 23 mm	8 (8.0)	4 (7.1)	4 (9.1)	–
Evolut R 26 mm	44 (44.0)	17 (30.4)	27 (61.4)	–
Evolut R 29 mm	33 (33.0)	24 (42.9)	9 (20.5)	–
Evolut R 34 mm	15 (15.0)	11 (19.6)	4 (9.1)	–
Pre-TAVR balloon valvuloplasty	33 (33.0)	16 (28.6)	17 (38.6)	0.39
Post-TAVR balloon valvuloplasty	49 (49.0)	28 (50.0)	21 (47.7)	0.98

Values are reported as the mean and sd or number and percentage (%).

BMI, body mass index; BSA, body surface area; COPD, chronic obstructive pulmonary disease; IDDM, insulin-dependent diabetes mellitus; MI, myocardial infarction; NIDDM, non-insulin-dependent diabetes mellitus; NYHA, New York Heart Association; PCI, percutaneous coronary intervention; TIA, transient ischaemic attack.

^a^Refer to hypoattenuated lesions at the level of the leaflet, and or anatomic sinus and or subvalvular component of the prosthesis.

^b^Body surface area was calculated with the Du Bois formula: 0.007 × weight^0.425^ × height^0.725^.

^c^The BMI was calculated as the weight in kilograms divided by the square of the height in metres.

Data were available for the entire cohort.

### Valvular and perivalvular hypoattenuated lesions

**Figure 4:**
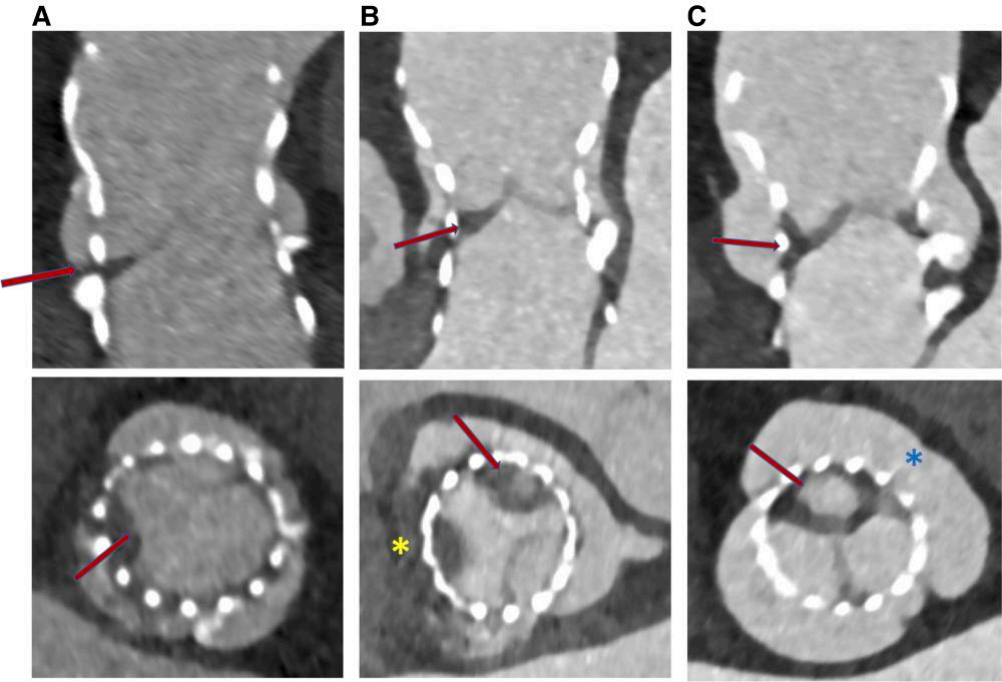
Example of hypoattenuated leaflet thickening visualised with multi-detector computed tomography in the long and short axes (red arrows); (*A*) hypoattenuated leaflet thickening < 25%; (*B*) hypoattenuated leaflet thickening 25–50%; (*C*) hypoattenuated leaflet thickening > 75%. Asterisk in the (*B*) panel refers to association with sinus thrombosis; asterisk in the (*C*) panel refers to bicuspid valve.

**Figure 5:**
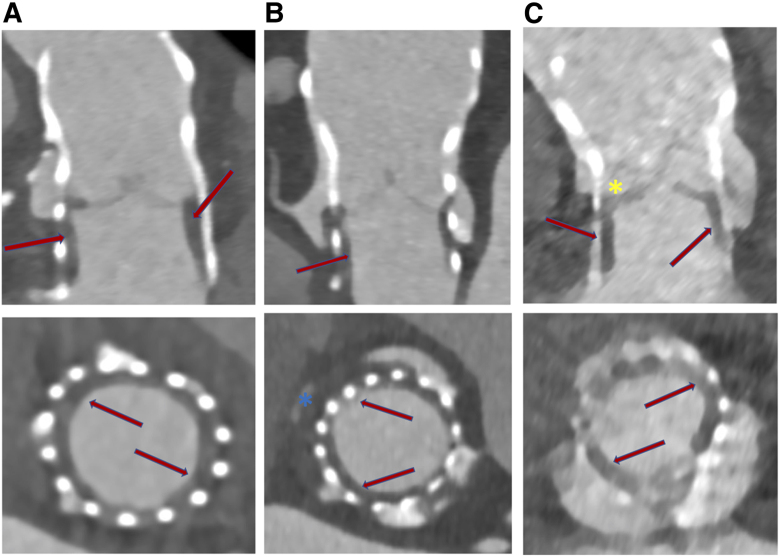
(*A-C*) Subvalvular thrombosis visualised with multi-detector computed tomography in long and short axes (red arrows). Asterisk in the (*B*) panel indicates association with anatomic sinus thrombosis. Asterisk in the (*C*) panel indicates concomitant leaflet thrombosis.

Among the 100 patients with evaluable MDCT scans at 6 months, 44 (44%) had thrombus at any aortic valve complex. Leaflet thrombosis was detected in 18 (18%) patients; 6 (6%) had at least one leaflet with severe thrombosis (*Figure 4A–C*). Breakdown of the extent of HALT and RELM is presented in *Table 2*; only two patients had severe RELM of at least one leaflet. Subvalvular thrombosis with partial or complete circumferential involvement of the prosthesis inner skirt was diagnosed in 23 patients (23%) (*Figure 5A–C*).

**Table 2:** Hypoattenuated lesions at valvular and perivalvular level

*n* = 100	
Patient with leaflet thrombosis, *n* (%)^a^	18 (18)
Involvement of a single leaflet	7 (7)
Involvement of two leaflets	4 (4)
Involvement of three leaflets	7 (7)
Hypoattenuated lesions leaflet *n* (%)	
HALT≤25%	9 (9)
HALT > 25–50%	17 (17)
HALT > 50–75%	2 (2)
HALT > 75%	8 (8)
Restricted leaflet movement (%)	
RELM≤25%	16 (16)
RELM > 25–50%	18 (18)
RELM > 50–75%	2 (2)
RELM > 75%	0
Patient with sinus thrombosis, *n* (%)	24 (24)
Involvement of one sinus	17 (17)
Involvement of two sinuses	5 (5)
Involvement of three sinuses	2 (2)
Hypoattenuated lesions anatomic sinus *n* (%)	
LCC	10 (10)
RCC	8 (8)
NCC	25 (25)
Hypoattenuated lesions subvalvular—prosthesis inner skirt *n* (%)	23 (23)

Values are reported as mean and Sd, or number and percentage (%).

HALT, hypoattenuated leaflet thickening; LCC, left coronary cusp; NCC, non-coronary cusp; RCC, right coronary cusp; RELM, restricted leaflet movement.

^a^One patient may have more than one of subclinical leaflet thrombosis/HALT simultaneously or thrombus formation in more sinuses.

**Figure 6:**
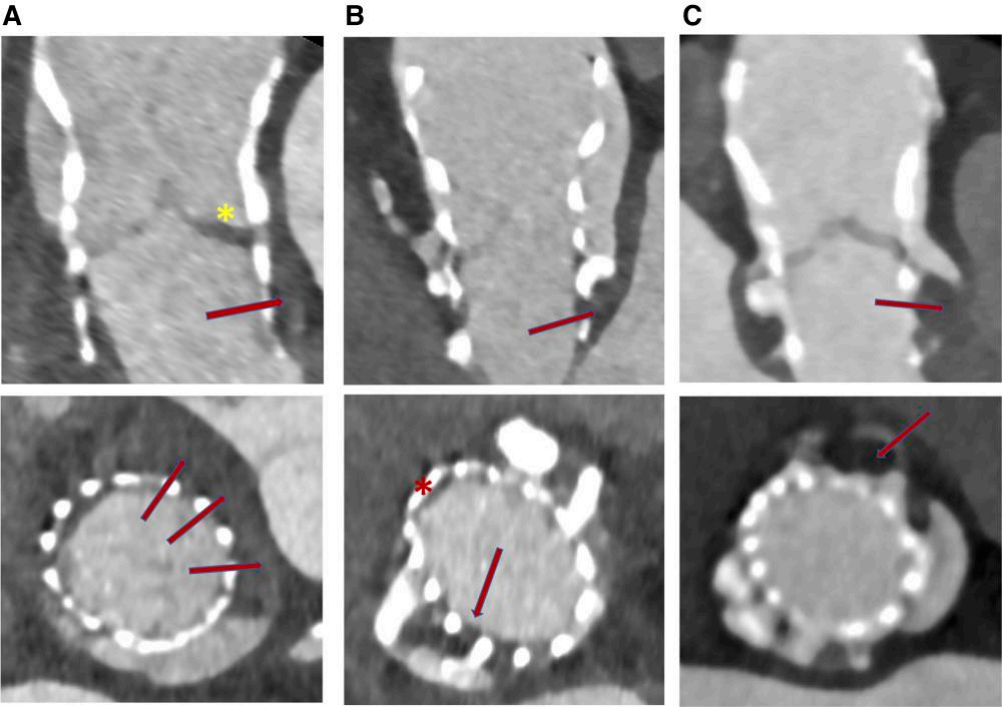
(*A-C*) Anatomic sinus thrombosis visualised with multi-detector computed tomography in long and short axes (red arrows). Asterisk in panel (*A)* indicates association with leaflet thrombosis. Asterisk in panel (*B*) indicates high eccentricity index.

Thrombosis of the anatomic sinus was detected in 24 patients (24%) and was more prevalent in the non-coronary sinus (*Figure 6A–C*). Two patients had thrombus stratification in all three anatomic sinuses. In three patients, the thrombus affected all components (i.e. the leaflet, anatomic sinus, and subvalvular level). Baseline clinical characteristics, including the use of anticoagulants at follow-up, were not significantly different between the two groups (*Table 1*). With regard to the peri-procedural characteristics, smaller valve sizes were implanted in the group with thrombosis compared with the group with no thrombosis.

### Device–host multi-detector computed tomography characteristics

**Figure 7:**
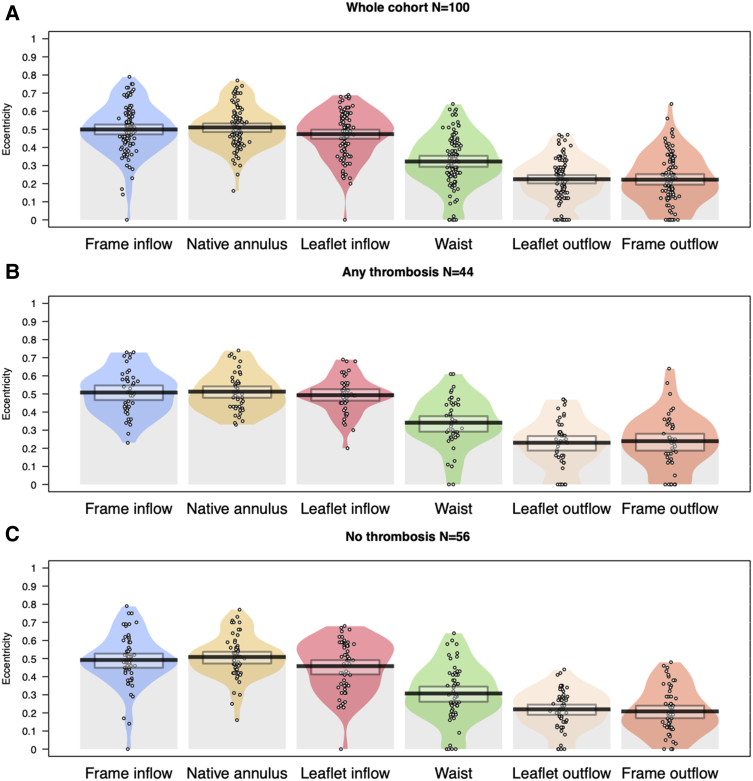
(*A-C*) Eccentricity index calculated at six prespecified prosthesis levels. (*A*) all cohort, *n* = 100, mean eccentricity index: 0.38 ± 0.08; (*B*) group with thrombosis, *n* = 44, mean eccentricity index 0.39 ± 0.08; (*C*) group with no thrombosis, *n* = 56, mean eccentricity index: 0.37 ± 0.08.

Are reported in *Table 3*. The highest eccentricity was observed at the annulus level (index: 0.51 ± 0.11), while the lowest was observed at the outflow level (index: 0.22 ± 0.14). The eccentricity index constantly decreased from the native annulus to the frame outflow (*Figure 7A*). There was also a constantly higher yet not significant degree of eccentricity in the group with valvular and perivalvular thrombosis compared with the group with no thrombosis (*Figure 7B and C*); implantation depth was significantly lower in the group with thrombosis than in the group with no thrombosis (5.23 ± 2.25 vs. 6.46 ± 3.60 mm, *P* = 0.05). Bicuspid valves were more frequent in the group with thrombosis than in that without thrombosis (29.5 vs. 5.4%, *P* = 0.003). Importantly, higher mean eccentricity was noted in the bicuspid group than in the rest of the population (*P* = 0.02; Supplementary material online, *Tables S1* and *S2*).

**Table 3:** Device–host multi-detector computed tomography characteristics

	Overall cohort	Valvular and/or perivalvular thrombosis^a^		*P*-value
	*n* = 100			
		No	Yes	
		*n* = 56	*n* = 44	
Eccentricity, mean ± Sd	0.38 (0.08)	0.37 (0.08)	0.39 (0.08)	0.234
Frame inflow	0.50 (0.14)	0.49 (0.15)	0.51 (0.12)	0.57
Native annulus	0.51 (0.11)	0.51 (0.12)	0.51 (0.11)	0.86
Leaflet inflow	0.47 (0.13)	0.46 (0.14)	0.49 (0.10)	0.17
Prosthesis constrained—waist	0.32 (0.15)	0.31 (0.16)	0.34 (0.14)	0.26
Leaflet outflow	0.22 (0.12)	0.22 (0.11)	0.23 (0.13)	0.66
Frame outflow	0.22 (0.14)	0.21 (0.13)	0.24 (0.15)	0.32
Asymmetric leaflet expansion, degree, mean±Sd	11.28 (6.18)	10.39 (5.26)	12.41 (7.08)	0.10
Commissural misalignment, *n* (%)^b^	22 (22)	10 (10)	12 (12)	0.51
Implantation depth, mm, mean±Sd	5.92 (3.13)	6.46 (3.60)	5.23 (2.25)	0.05
Valve-to-coronary length, mm, mean±SD				
Left coronary	5.94 (1.91)	5.84 (1.86)	6.06 (2.0)	0.56
Right coronary	5.47 (2.10)	5.60 (2.34)	5.30 (1.75)	0.48
Non-coronary	5.87 (1.99)	5.79 (1.67)	5.88 (2.02)	0.55
Bicuspid valve	16 (16)	3 (5.40)	13 (29.50)	0.003
Type 0	–	0	2 (4.54)	–
Type I/II	–	2 (3.57)	12 (27.27)	–

Values are reported as mean and Sd, or number and percentage (%).

LCC, left coronary cusp; NCC, non-coronary cusp; RCC, right coronary cusp.

^a^Refer to hypoattenuated lesions at the level of the leaflet, and or anatomic sinus and or subvalvular component of the prosthesis.

^b^Defined as at least moderate.

### Predictors of valvular and perivalvular thrombosis

Bicuspid valve was the predictor with highest association with hypoattenuated lesions (LASSO coefficient 0.35, 95%, CI 0.21–0.68) (Supplementary material).

### Prosthesis function analysis

**Figure 8:**
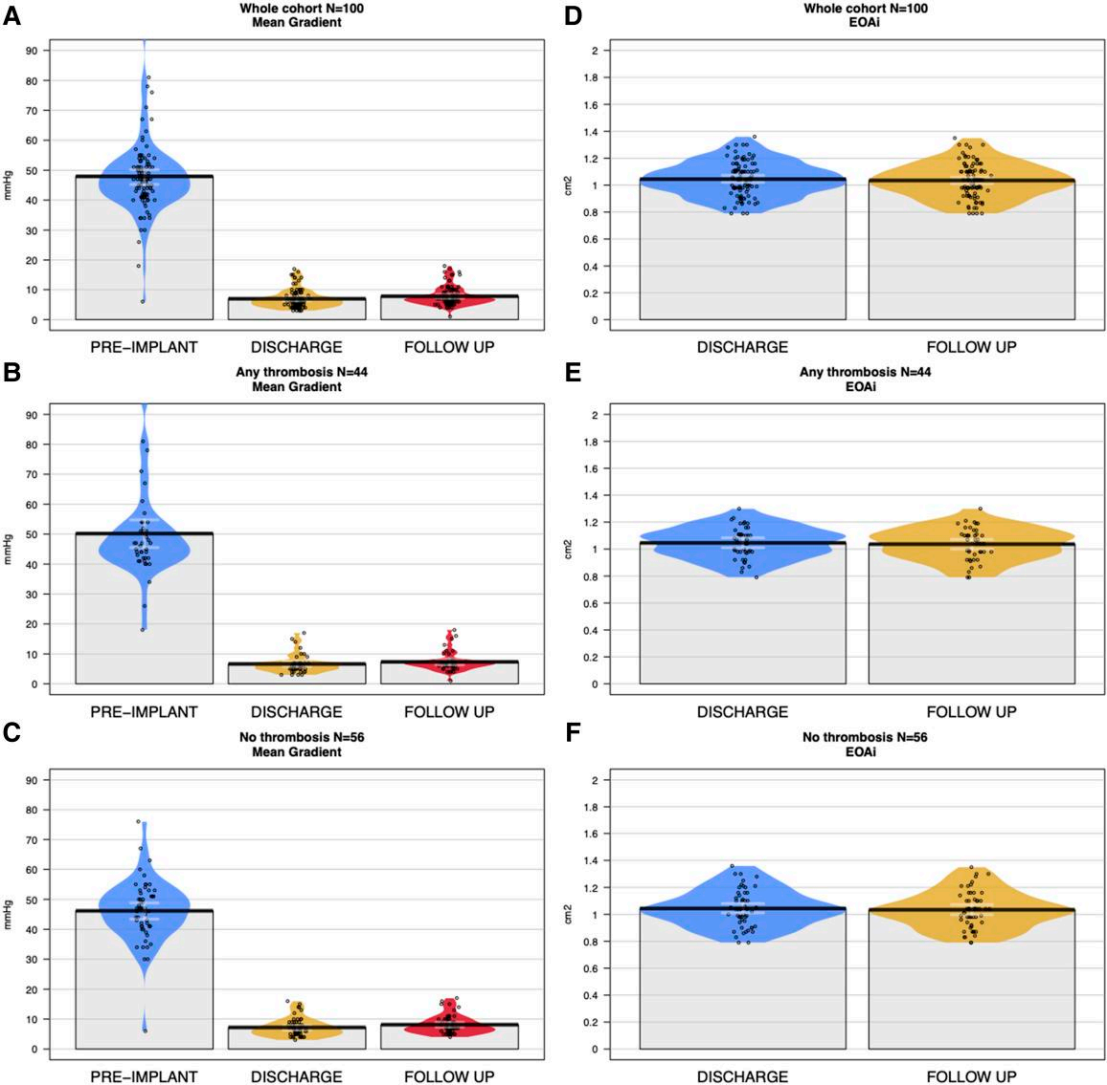
Left panel: mean gradient (mmHg) pre-implantation, at hospital discharge and at follow-up in the (*A*) overall cohort, 47.99 ± 13.18, 6.96 ± 3.05, 7.12 ± 3.59, (*B*) group with thrombosis, 50.33 ± 15.55, 6.60 ± 3.08, 6.97 ± 4.16 and (*C*) group without thrombosis 46.10 ± 10.69, 7.10 ± 3.02, 7.23 ± 3.14. Right panel effective orifice area indexed (cm^2^/body surface area) at hospital discharge and at follow-up in the (*D*) overall cohort, 1.03 ± 0.15, 1.03 ± 0.13, (*E*) group with thrombosis 1.04 ± 0.16, 1.04 ± 0.12, and (*F*) group without thrombosis 1.03 ± 0.16, 1.03 ± 0.14(see Supplementary material online, *Table S2*).

Echocardiographic characteristics at baseline, discharge and 6 months follow-up are shown in Supplementary material online, *Table S3*. The mean aortic valve gradient after TAVR remained low, with no significant difference between the groups with and without thrombosis (6.97 ± 4.16 vs. 7.23 ± 3.14 mmHg, *P* = 0.73) (*Figure 8A–C*). In the two patients with severe RELM (> 50%, one leaflet), the mean gradients were 14 and 20 mmHg, respectively, and the latter was the only patient in whom an HSVD diagnosis was made because of a concomitant decrease in EOA ≥ 0.3 cm^2^. At 6 month follow-up, effective orifice area indexed was similar between the groups with and without thrombosis (1.04 ± 0.12 vs. 1.03 ± 0.14 cm^2^, *P* = 0.90), with no significant changes compared to discharge (*Figure 8D–F*). Bioprosthetic valve dysfunction diagnosis (haemodynamic and non-structural definition) was reached in seven (15.9%) and ten (17.9%) patients in the groups with and without thrombosis respectively (*P* = 0.99). The subgroup analysis comparing patients with leaflet thrombosis vs. the rest of the population is provided in the Supplementary material/Supplementary material online, *Figures S5* and *S6* and did not show significant differences in terms of incidence of HSVD and bioprosthetic valve dysfunction.

### Clinical outcome

The clinical outcomes are provided in Supplementary material online, *Table S4*. Seven patients were discharged on oral anticoagulants; during follow-up two more patients were also started on anticoagulants for episodes of atrial fibrillation. At the time of MDCT scan 18 patients were receiving dual antiplatelet therapy. One episode of acute coronary syndrome caused by a fresh thrombus at the level of the right coronary artery treated with coronary angioplasty occurred 2 days after the index procedure. Later, MDCT revealed the presence of a thrombus at the level of the right and non-coronary sinus (*Video 1*). Overall, five episodes of TIA occurred during follow-up (two in the group with thrombosis), with no sequelae; there were three episodes of re-hospitalisation for heart failure. There was no difference in terms of the combined end-point neurological events/re-hospitalization (valvular and non-valvular related) between the groups with and without thrombosis (HR: 0.86, 95% CI: 0.24–3.06, log-rank test *P* = 0.82; Supplementary material online, *Figure S7*).

## Discussion

With this study based on the analysis of 100 MDCT scans and 2D TTE at the 6 month follow-up, we were able to demonstrate the following (*Graphical abstract*):

- Hypoattenuated lesions, suggesting the presence of thrombus at the leaflet, anatomic sinus, and subvalvular level, were common findings in the context of self-expandable prostheses (i.e. Evolut R).
- Among the MDCT host-prosthesis factors analysed, the native bicuspid valve showed the strongest association with thrombus at any valvular complex, which can be related to the higher level of eccentricity/asymmetrical expansion in this group than in the rest of the population.
- Hypoattenuated lesions were generally benign, with no haemodynamic or clinical consequences.

The Evolut R prosthesis has a 13–14 mm length inner skirt that extends from the frame inflow to the leaflet inflow.^17^ This section is manufactured with a self-expandable nitinol frame covered by pliable porcine pericardial tissue with a width of 0.2 mm. It has been reported by the manufacturer a ‘stable and mature’ pericardial tissue growth at 90 days post-implant as a thin and even layer on the inner side of the skirt.^17^ However, while this pericardial tissue is supposed to be characterized by a low inflammatory response, it may display a phenomenon of hypoattenuated thickening, analogous to the leaflets.^5,6^

The ‘anatomic sinus’ refers to the artificially created space formed after TAVR that occupies the region situated between the prosthesis cage and the aortic wall.^7^ The anatomic sinus space accommodates the native calcific aortic leaflets following prosthesis expansion and it is believed to serve as a site of blood stasis, which may result in the formation of thrombus.^7^

No other research has evaluated the prevalence and determinants of valvular and perivalvular thrombus specifically in the context of self-expandable prostheses.

The left thickening-immobility substudy was the prespecified component of the Evolut Low-Risk trial and reported an SLT/HALT frequency of 30.9% at 1 year^12^ in a population without anticoagulant therapy. However, the analysis was limited to the leaflet, and did not evaluate perivalvular thrombosis. Also, no predictors were found to be associated with SLT/HALT at multivariable analysis.

Fukui *et al.*^8^ analysed MDCT scans from 213 patients who had been implanted with Evolut R/PRO; even in this case the study was limited to the leaflets and SLT/HALT was present in 16% of the prostheses assessed. The occurrence of SLT/HALT was associated to prosthesis deformation, leaflet asymmetric expansion and smaller anatomic sinus. However, this study had a short follow-up period of only 30 days.

The first comprehensive analysis of thrombosis at any valvular complex was carried out by Koo, who described subvalvular hypoattenuated thickening in a mixed cohort of patients treated with balloon and self-expandable prosthesis,^5^ with a median duration from TAVR to MDCT of 17.5 days. These outcomes were supported by the results of a recent prespecified, dedicated MDCT substudy of a clinical trial involving balloon-expandable valves which demonstrated that nearly 43.1% of the sample displayed thrombus in the aortic valve complex.^4^ In accordance with these findings, 44% of the patients in our case series exhibited thrombus in any aortic valve complex.

Other aspects of our study warrant further discussion.

- It is not entirely clear why native bicuspid valve is associated with the development of hypoattenuated lesions. As demonstrated in our series, abnormal expansion or eccentricity of the prosthesis can arise relative to the native bicuspid valve, potentially leading to the development of hypoattenuated lesions. Importantly, the bicuspid valve may be associated with altered haemodynamics and regional aortic wall shear stress, which can predispose to thrombus formation.^18^
- Among the analysis of prosthesis-host interaction characteristics, we were able to demonstrate that eccentricity decreases from the native annulus to the frame outflow, and this arrangement is called ‘stent-frame decoupling’ and is intended to mitigate the influence of ellipticity at the valve level following deployment.^19^
- Implantation depth significantly differed between the groups with and without thrombosis; however, this disparity may have been impacted by the method used to measure it. Moreover, it is worth mentioning that the VARC-3 criteria do not propose a particular technique for assessing this parameter.^9^
- The incidence of haemodynamic structural valve dysfunction did not differ between groups with or without thrombosis; although hypoattenuated lesions are frequently present after TAVR, this condition is typically silent during echocardiography.

### Study limitation

The relatively limited patient population in this study precluded the execution of a more comprehensive analysis that incorporated additional MDCT predictors (i.e. calcium score or volumetric assessment of the anatomic sinus), or other important clinical components such as oral anticoagulants. However, LASSO regression was used to mitigate overfitting; additionally, the number of events of interest (i.e. any hypoattenuated lesions) was very frequent, accounting for 44% of the sample. The possibility in some cases that hypoattenuated lesions may have been incorrectly adjudicated as thrombus formation may not be fully ruled out.

Only two cases of Type 0 bicuspid valve were included in our case series; hence, we could not evaluate the frequency of valvular and perivalvular thromboses in these patients. The results of our study may not accurately reflect the broader population, as the patients included in our analysis were generally considered low risk. Additionally, many patients with atrial fibrillation with indications for oral anticoagulants were excluded because of poorly controlled heart rhythm. Echocardiograms were obtained by different operators; hence, a certain degree of inter-operator variability cannot be ruled out. Finally, only Evolut R was included in the analysis, hence more studies are needed to validate these findings in the context of the newer prostheses.

## Conclusions

This study confirms that thrombus stratification often affects the leaflet, the inner skirt and the anatomic sinus of the Evolut R prosthesis, and native bicuspid valve showed the strongest association with valve thrombosis. Clinical and haemodynamic consequence of thrombosis at any complex were generally benign, however further studies with longer MDCT follow-up are needed to determine the fate of valvular and perivalvular thrombosis.

## Lead author biography


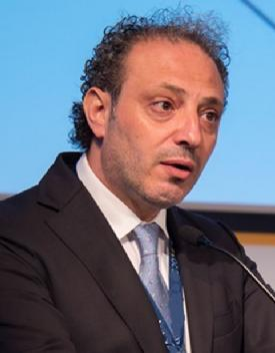


Khalil Fattouch is the lead consultant cardiac surgeon at GVM Care & Research, Maria Eleonora Hospital, Palermo, Italy. He is currently Professor in Cardiac Surgery at Kore University, Enna, Italy and co-founder and director of the Mitral Academy. His clinical practice and research focuses on minimally invasive valve surgery. He has given expert presentations at national and international meetings to share his knowledge. He currently serves as the Editor, co-editor, and scientific committee member of several international meeting. Throughout his academic career, he has authored more than 120 peer-reviewed journal articles along with numerous book chapters. Along with his team, he recently secured a European grant to investigate the predictors of early prosthesis dysfunction following transcatheter and surgical aortic valve replacement (www.endotavi.it).

## Supplementary Material

oeae085_Supplementary_Data

## Data Availability

The data underlying this article will be shared on reasonable request to the corresponding author.
