# Supplementary material for: Valvular and perivalvular thrombosis following self-expandable aortic valve replacement: analysis of 100 multi-detector computed tomography scans
Source: Eur Heart J Open. 2024 Oct 15;4(5):oeae085. doi: 10.1093/ehjopen/oeae085 (PMC11648950; doi:10.1093/ehjopen/oeae085)
Supplement: oeae085_Supplementary_Data [file oeae085_Supplementary_Data.docx]

**SUPPLEMENTARY MATERIAL:**

**PROTOCOL and SUPPLEMENTARY RESULTS**

| **Principal Investigators & Research Team Contact Details** | | | |
| --- | --- | --- | --- |
| **Principal investigator (PI)** |  | **Principal investigator (PI)** |  |
| Khalil Fattouch  Policlinico Paolo Giaccone  Maria Eleonora Hospital, GVM Care&Research  Viale della Regione Siciliana Nord Ovest, 1571, 90135 Palermo PA, Italy  Contact:  [khalilfattouch@hotmail.it](mailto:khalilfattouch@hotmail.it)  Tel: +39 091 6981111 |  | Marco Moscarelli  Maria Eleonora Hospital, GVM Care&Research  Viale della Regione Siciliana Nord Ovest, 1571, 90135 Palermo PA, Italy  Contact:  [m.moscarelli@imperial.ac.uk](mailto:m.moscarelli@imperial.ac.uk) |  |
| Dariusz Dudek  Department of Cardiology and Cardiovascular Interventions, Jagiellonian University Hospital  Macieja Jakubowskiego 2  30-688 Krakow, Poland  Contact  [mcdudek@cyfronet.pl](mailto:mcdudek@cyfronet.pl) |  | Adriana Zlahoda-Huzior  AGH University of Science and Technology, Department of Measurement & Electronics, Krakow, Poland  Contact  [adrianazlahoda@gmail.com](mailto:adrianazlahoda@gmail.com) |  |
| Patrizio Lancellotti  University of Liège Hospital, GIGA Institute, Department of Cardiology, CHU Sart Tilman, Liège, Belgium  Contact  [plancellotti@chuliege.be](mailto:plancellotti@chuliege.be) |  |  |  |
| Francesco Cappello  Istituto di Anatomia Umana e Istologia Dipartimento di  Biomedicina, Neuroscience e Diagnostica avanzata (BIND)  Università degli Studi di Palermo /Scientific Director (Honorific), Euro-  Mediterranean Institute of Science and Technology, Palermo, Italy  Contact:  francap@hotmail.com |  | Valentina Di Felice  Istituto di Anatomia Umana e Istologia Dipartimento di  Biomedicina, Neuroscience e Diagnostica avanzata (BIND) and Euro-  Mediterranean Institute of Science and Technology, Palermo, IEMEST, Italy  Università degli Studi di Palermo  Contact:  Valentina.difelice@unipa.it |  |
| **Research team / Main collaborator** |  |  |  |
| Rosa Prestera  Policlinico Paolo Giaccone  Via del Vespro, 129, 90127 Palermo PA, Italy  Dept of Cardiology  Contact:  [rosapresetera@gmail.com](mailto:rosapresetera@gmail.com) |  | Claudia De Pasquale  Maria Eleonora Hospital, GVM Care&Research  Viale della Regione Siciliana Nord Ovest, 1571, 90135 Palermo PA, Italy  Contact:  [cdipasquale@gvmnet.it](mailto:cdipasquale@gvmnet.it) |  |
| Francesco Violante  Maria Eleonora Hospital, GVM Care&Research  Viale della Regione Siciliana Nord Ovest, 1571, 90135 Palermo PA, Italy  Dept. Of Radiology  Contact:  [violanterx@libero.it](mailto:violanterx@libero.it) |  | Sabrina Maria Milo  Maria Eleonora Hospital, GVM Care&Research  Viale della Regione Siciliana Nord Ovest, 1571, 90135 Palermo PA, Italy  Dept. Of Radiology  Contact:  [smmilo@gvmnet.it](mailto:smmilo@gvmnet.it) |  |
| Giulia Sollami  Maria Eleonora Hospital, GVM Care&Research  Viale della Regione Siciliana Nord Ovest, 1571, 90135 Palermo PA, Italy  Dept. Of Radiology  Contact:  [giuliasollami@hotmail.it](mailto:giuliasollami@hotmail.it) |  | Vincenzo Pernice  Maria Eleonora Hospital, GVM Care&Research  Viale della Regione Siciliana Nord Ovest, 1571, 90135 Palermo PA, Italy  Contact:  [vpernice@gvmnet.it](mailto:vpernice@gvmnet.it) |  |
| Emanuele Lentini  Maria Eleonora Hospital, GVM Care&Research  Viale della Regione Siciliana Nord Ovest, 1571, 90135 Palermo PA, Italy  Contact:  emanuelentini89@gmail.com |  | Francesca Trizzino  Maria Eleonora Hospital, GVM Care&Research  Viale della Regione Siciliana Nord Ovest, 1571, 90135 Palermo PA, Italy  Contact:  [ftrizzino@gvmnet.it](mailto:ftrizzino@gvmnet.it) |  |
| **Medical statistician** |  |  |  |
| Francesca Fiorentino  King's College London as Senior Lecturer in Clinical Trials Statistics within the Nightingale-Saunders Clinical Trials & Epidemiology Unit (King’s CTU), London, UK  Contact:  [francesca.fiorentino@kcl.ac.uk](mailto:francesca.fiorentino@kcl.ac.uk) |  | Marco Moscarelli  Maria Eleonora Hospital, GVM Care&Research  Viale della Regione Siciliana Nord Ovest, 1571, 90135 Palermo PA, Italy  Contact:  [m.moscarelli@imperial.ac.uk](mailto:m.moscarelli@imperial.ac.uk) |  |

| **Table of contents** |  |
| --- | --- |
| **1. Study population: inclusion and exclusion criteria** |  |
| **2. MDCT protocol** |  |
| **SUPPLEMENTARY RESULTS** |  |
| **~~Lasso regression~~** |  |
| **Bicuspid valve breakdown** |  |
| **R-code** |  |
| **Supplementary tables** |  |
| Supplementary table 1 | Characteristics of the Patients at Baseline and Procedural Details According to Bicuspid vs. no-Bicuspid group |
| Supplementary table 2 | Device-Host MDCT Characteristics Bicuspid vs. no-Bicuspid group |
| Supplementary table 3 | Echocardiographic Characteristics According to Thrombus at Any Valve Complex |
| Supplementary table 4 | Clinical outcome |
| **Supplementary figure** |  |
| Supplementary figure 1 | Commissural Alignment |
| Supplementary figure 2 | Leaflet Expansion |
| Supplementary figure 3 | Implantation Depth |
| Supplementary figure 4 | Valve to coronary and to non-coronary sinus distance |
| **Subgroup analysis** | HALT/SLT vs. rest of the population |
| Supplementary Figure 5 | Mean gradient at follow-up |
| Supplementary Figure 6 | Effective orifice area |
| **Survival analysis** |  |
| Supplementary figure 7 | Time to event analysis |

**1. Study population: Inclusion and Exclusion criteria**

Patient with severe symptomatic aortic stenosis with indication to TAVR (transfemoral route (TF)).

Inclusion criteria:

All patients with severe symptomatic aortic stenosis implanted with Evolut R.

Exclusion criteria:

1. Valve in valve procedure
2. Aneurysm of the Valsalva and / or ascending aorta
3. Previous root replacement
4. Pure aortic regurgitation
5. Other significant valve disease
6. Implanted devices other than Evolut - Medtronic (i.e., Abbott – Portico / Balloon expandable)
7. Moderate to severe renal failure (glomerular filtration rate <40)
8. Impossibility to keep the heart rate below 60 bpm at the time of MDCT scan (i.e., poorly controlled atrial fibrillation)
9. Allergy to iodine contrast and other contra-indication to MDCT
10. Patients unwilling to participate to the study or uncapable to sign consent form or participating to another study.

The use of dual antiplatelet therapy, vitamin K antagonists (VKAs), or new oral anticoagulants (NOACs) does not represent an exclusion criterion.

**2. MDCT protocol**

*- Images acquisition*

Contrast – enhanced MDCT scans were performed using Siemens Healthcare GmbH SOMATOM Drive (VB 20, 2019).

An antecubital intravenous access site was placed (at least a 20 Gauge cannula) to deliver iodinated contrast (between 50 and 100 ml). ECG-gated synchronised imaging was performed to achieve full cardiac cycle acquisition. Image reconstruction was performed in 5–10% increments of the R-R interval (equating to 10–20 separate phases). Slice thickness was set to 0.5 mm to improve diagnosis accuracy. The standard tube potential for the procedure was maintained between 100 and 120 kV or higher for obese patients (140 kV) or for patients with other intracardiac devices.

Care was taken to perform image acquisition while maintaining a heart rate below 70 beats/min as higher rate may result in image artefacts. Poorly controlled atrial fibrillation represented an exclusion criterion. Beta-blockers (i.v.) could be used to reduce the heart rate. Images were acquired during peak arterial enhancement in the ascending aorta. This was performed during contrast bolus tracking with automatic triggers once the aortic enhancement reaches >300 Hounsfield units (HU).

**SUPPLEMENTARY RESULTS**

**~~LASSO REGRESSION~~**

~~For the R code below, the following packages were used:~~

~~1) glmnet: ‘Lasso and Elastic-Net Regularized Generalized Linear Models’~~

~~2) HDCI: ‘High Dimensional Confidence Interval Based on Lasso and Bootstrap’.~~

**~~R code:~~**

**
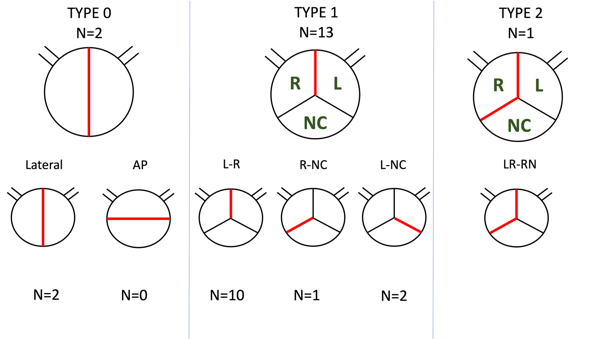
**

Native bicuspid valve breakdown according to Sievers classification. AP: antero-posterior. NC: non coronary. L: left. R: right.

**SUPPLEMENTARY TABLES**

| **Suppl. Table 1. Characteristics of the Patients at Baseline and Procedural Details According to Bicuspid vs. no-Bicuspid** | | | | | |
| --- | --- | --- | --- | --- | --- |
|  | **Overall cohort**  **N=100** | **Bicuspid** | |  |  |
|  |  | **NO**  **N=84** | **YES**  **N=16** | **p-value** |  |
| Age, yr | 78.2 (5.8) | 77.9 (5.9) | 79.6 (5.3) | 0.31 |  |
| BSA^∫^, m^2^ | 1.7 (0.22) | 1.7 (0.2) | 1.6 (0.1) | 0.001 |  |
| BMI^χ^ | 27.8 (5.0) | 28.51 (5) | 24.62 (3.4) | 0.004 |  |
| Male sex, no. (%) | 53 (53.0) | 45 (53.6) | 8 (50) | 0.99 |  |
| Euroscore II, % | 2.03 (1.02) | 2.06 (1.09) | 1.84 (0.51) | 0.43 |  |
| Low flow – low gradient, n (%) | 11 (11.0) | 10 (11.9) | 1 (6.2) | 0.82 |  |
| NYHA functional class III-IV, no. (%) | 13 (13.0) | 9 (10.7) | 4 (25) | 0.24 |  |
| NIDDM/IDDM, no. (%) | 30 (30.0) | 27 (32.1) | 3 (18.8) | 0.43 |  |
| Hypertension, no. (%) | 98 (98.0) | 82 (97.6) | 16 (100) | 0.99 |  |
| COPD, no. (%) | 18 (18.0) | 14 (16.7) | 4 (25) | 0.66 |  |
| Previous stroke/TIA, no. (%) | 4 (4.0) | 2 (2.4) | 2 (12.5) | 0.23 |  |
| Previous PCI, no. (%) | 22 (22.0) | 19 (22.6) | 3 (18.8) | 0.98 |  |
| Previous cardiac surgery, no. (%) | 9 (9.0) | 9 (10.7) | 0 (0) | 0.37 |  |
| Previous MI, no. (%) | 17 (17.0) | 15 (17.9) | 2 (12.5) | 0.87 |  |
| Coronary artery disease, no. (%) | 28 (28.0) | 25 (29.8) | 3 (18.8) | 0.55 |  |
| Serum creatinine, mg/dl | 0.8 (0.2) | 0.8 (0.2) | 0.8 (0.2) | 0.77 |  |
| Pre-existing pacemaker or defibrillator, no. (%) | 11 (11.0) | 10 (11.9) | 1 (6.2) | 0.82 |  |
| History of right bundle-branch block, no. (%) | 3 (3.0) | 2 (2.4) | 1 (6.2) | 0.97 |  |
| Treatment with vitamin K antagonist, no. (%) | 2 (2.0) | 2 (2.4) | 0 (0) | 0.99 |  |
| Treatment with direct oral anticoagulant, no. (%) | 5 (5.0) | 5 (6) | 0 (0) | 0.70 |  |
| Dual antiplatelets therapy, no. (%) | 23 (23.0) | 15 (26.8) | 8 (18.2) | 0.43 |  |
| *Procedural characteristics:* |  |  |  |  |  |
| Valve size |  |  |  | 0.32 |  |
| Evolut R 23 mm | 8 (8.0) | 8 (9.5) | 0 (0) |  |  |
| Evolut R 26 mm | 44 (44.0) | 34 (40.5) | 10 (62.5) |  |  |
| Evolut R 29 mm | 33 (33.0) | 29 (34.5) | 4 (25) |  |  |
| Evolut R 34 mm | 15 (15.0) | 13 (15.5) | 2 (12.5) |  |  |
| Pre-TAVR balloon valvuloplasty | 33 (33.0) | 24 (28.6) | 9 (56.2) | 0.06 |  |
| Post-TAVR balloon valvuloplasty | 49 (49.0) | 37 (44) | 12 (75) | 0.04 |  |
| Values are reported as the mean and standard deviation (SD) or number and percentage (%). BMI: body mass index. BSA: body surface area. COPD: chronic obstructive pulmonary disease. IDDM: insulin-dependent diabetes mellitus. MI: myocardial infarction. NIDDM: non-insulin dependent diabetes mellitus. NYHA: New York Heart Association. PCI: percutaneous coronary intervention. TIA: transient ischemic attack.  ^∫^ Body surface area was calculated with the Du Bois formula: 0.007xWeight^0.425^xHeight^0.725^.  ^χ^The body mass index was calculated as the weight in kilograms divided by the square of the height in meters.  Data was available for the entire cohort. | | | | | |

| **Suppl. Table 2. Device-Host MDCT Characteristics Bicuspid vs. no-Bicuspid** | | | | | |
| --- | --- | --- | --- | --- | --- |
|  | | **Overall cohort**  **N=100** | **Bicuspid** | |  |
|  | |  | **NO**  **N=84** | **YES**  **N=16** | **p-value** |
| Eccentricity, mean $\pm$ *SD* |  | 0.38 (0.08) | 0.37 (0.08) | 0.43 (0.09) | 0.005 |
| Frame inflow |  | 0.50 (0.14) | 0.49 (0.15) | 0.57 (0.13) | 0.023 |
| Native annulus |  | 0.51 (0.11) | 0.51 (0.11) | 0.57 (0.13) | 0.034 |
| Leaflet inflow |  | 0.47 (0.13) | 0.46 (0.13) | 0.53 (0.12) | 0.048 |
| Prosthesis constrained - waist |  | 0.32 (0.15) | 0.31 (0.15) | 0.40 (0.14) | 0.025 |
| Leaflet outflow |  | 0.22 (0.12) | 0.22 (0.12) | 0.26 (0.12) | 0.199 |
| Frame outflow |  | 0.22 (0.14) | 0.22 (0.13) | 0.24 (0.17) | 0.61 |
| Asymmetric leaflet expansion, degree, mean $\pm$ SD |  | 11.28 (6.18) | 11.35 (6.25) | 10.94 (5.94) | 0.81 |
| Implantation depth, mm, mean $\pm$ SD |  | 5.92 (3.13) | 6.04 (3.21) | 5.28 (2.66) | 0.37 |
| Valve to coronary length, mm, mean $\pm$ *SD* |  |  |  |  |  |
| Left coronary |  | 5.94 (1.91) | 5.85 (1.90) | 6.37 (1.98) | 0.32 |
| Right coronary |  | 5.47 (2.10) | 5.61 (2.11) | 4.74 (1.92) | 0.13 |
| Values are reported as mean and standard deviation (SD), or number and percentage (%). LCC: Left coronary cusp. MDCT: Multi detector computed tomography. NCC: Non coronary cusp. RCC: Right coronary cusp. | | | | | |

| **Suppl. Table 3. Echocardiographic Characteristics According to Thrombus at Any Valve Complex** | | | | | |
| --- | --- | --- | --- | --- | --- |
|  | **Overall cohort**  **N=100** | **Valvular and/or perivalvular thrombosis^∍^** | |  |  |
|  |  | **NO**  **N=56** | **YES**  **N=44** | **p-value** |  |
| *Baseline* |  |  |  |  |  |
| LVEF %, mean $\pm$ SD | 54.04 (10.31) | 53.02 (11.53) | 55.30 (8.53) | 0.28 |  |
| LVEDD mm, mean $\pm$ SD | 45.84 (7.28) | 46.55 (7.36) | 44.94 (7.16) | 0.29 |  |
| IVS mm, mean $\pm$ SD | 13.43 (2.47) | 13.32 (2.86) | 13.56 (1.90) | 0.64 |  |
| PWT mm, mean $\pm$ SD | 12.41 (2.28) | 12.53 (2.43) | 12.26 (2.09) | 0.56 |  |
| AVMG mmHg, mean $\pm$ SD | 47.99 (13.18) | 46.10 (10.69) | 50.33 (15.55) | 0.12 |  |
| AVPG mmHg, mean $\pm$ SD | 80.52 (74.76) | 82.69 (98.66) | 77.83 (23.13) | 0.75 |  |
| PASP mmHg, mean $\pm$ SD | 37.27 (10.89) | 37.53 (11.50) | 36.95 (10.22) | 0.80 |  |
| *At discharge* |  |  |  |  |  |
| LVEF %, mean $\pm$ SD | 55.21 (6.71) | 55.25 (7.10) | 55.23 (6.60) | 0.68 |  |
| LVEDD mm, mean $\pm$ SD | 45.74 (7.21) | 46.55 (7.35) | 44.90 (7.11) | 0.29 |  |
| IVS mm, mean $\pm$ SD | 13.42 (2.47) | 13.33 (2.86) | 13.01 (1.91) | 0.63 |  |
| PWT mm, mean $\pm$ SD | 12.40 (2.28) | 12.55 (2.42) | 12.20 (2.01) | 0.56 |  |
| AVMG mmHg, mean $\pm$ SD | 6.96 (3.05) | 7.1 (3.02) | 6.6 (3.08) | 0.35 |  |
| AVPG mmHg, mean $\pm$ SD | 12.36 (5.11) | 12.80 (5.59) | 12.80 (6.73) | 0.55 |  |
| PASP mmHg, mean $\pm$ SD | 37.01 (10.11) | 37.03 (11.40) | 36.84 (10.01) | 0.69 |  |
| EOA cm^2^ | 1.85 (0.29) | 1.85 (0.31) | 1.87 (0.41) | 0.91 |  |
| EOAi cm^2^ | 1.03 (0.15) | 1.03 (0.16) | 1.04 (0.16) | 0.93 |  |
| *At follow-up* |  |  |  |  |  |
| LVEF %, mean $\pm$ SD | 55.61 (6.81) | 55.35 (7.03) | 55.92 (6.60) | 0.69 |  |
| LVEDD mm, mean $\pm$ SD | 46.89 (20.50) | 48.40 (27.10) | 45.89 (4.09) | 0.58 |  |
| IVS mm, mean $\pm$ SD | 11.06 (1.33) | 11.30 (1.34) | 10.74 (1.28) | 0.16 |  |
| PWT mm, mean $\pm$ SD | 11.45 (5.90) | 10.96 (1.90) | 12.11 (8.81) | 0.53 |  |
| AVMG mmHg, mean $\pm$ SD | 7.12 (3.59) | 7.23 (3.14) | 6.97 (4.16) | 0.73 |  |
| AVPG mmHg, mean $\pm$ SD | 13.47 (6.10) | 13.81 (5.62) | 13.00 (6.75) | 0.53 |  |
| PASP mmHg, mean $\pm$ SD | 33.92 (6.98) | 33.86 (7.22) | 34.00 (6.77) | 0.94 |  |
| Prosthetic aortic valve regurgitation |  |  |  | 0.42 |  |
| None/Trace | 61 (61.0) | 31 (55.4) | 30 (68.2) |  |  |
| Mild | 30 (30.0) | 19 (33.9) | 11 (25.0) |  |  |
| Moderate | 9 (9.0) | 6 (10.7) | 3 (6.8) |  |  |
| Severe | 0 | 0 | 0 |  |  |
| EOA cm^2^ | 1.87 (0.38) | 1.86 (0.33) | 1.87 (0.40) | 0.92 |  |
| EOAi cm^2^ | 1.03 (0.13) | 1.03 (0.14) | 1.04 (0.12) | 0.90 |  |
| PPM^*^ n, (%) |  |  |  | 0.98 |  |
| Moderate | 8 (8) | 5 (8.9) | 3 (6.8) |  |  |
| Severe | 0 |  |  |  |  |
| Increased 10 mmHg gradient from discharge, n (%) | 1 (1%) | 0 | 1 (2.2%) |  |  |
| Endocarditis, n (%) | 0 |  |  |  |  |
| Clinical thrombosis, n (%) | 0 |  |  |  |  |
| HSVD n, (%) | 1 (1%) | 0 | 1 (2.2%) |  |  |
| BVD n, (%) | 17 (17.0) | 10 (17.9) | 7 (15.9) | 0.76 |  |
| Values are reported as the mean and standard deviation (SD) or number and percentage (%). AVMG: Aortic valve peak gradient. AVMG: Aortic valve mean gradient. EOAi: Effective orifice area indexed. IVS: Interventricular septal thickness. LVEDD: Left ventricle end diastolic diameter. LVEF: Left ventricle ejection fraction. PASP: Pulmonary artery systolic pressure. PWT: Posterior wall thickness. BVD: Bioprosthetic valve dysfunction (according to VARC-3 criteria, that include hemodynamic and non-structural definition).  ^∍^ Include: leaflets, subvalvular and anatomic sinus hypoattenuated lesions.  ^*^Defined according to the Valve Academic Research Consortium-3 definition as follows: indexed effective orifice area (cm^2^/m^2^) for BMI <30: moderate=0.85-0.66 cm^2^/m^2^ and severe = $\leq$0.65 cm^2^/m^2^ and indexed effective orifice area (cm^2^/m^2^) for BMI $\geq$30: moderate= 0.70-0.56 cm^2^/m^2^ and severe = $\leq$0.55 cm^2^/m^2^. | | | | | |

| **Suppl. Table 4. Clinical outcome** | | | | | |
| --- | --- | --- | --- | --- | --- |
|  | **Overall cohort**  **N=100** | **Valvular and/or perivalvular thrombosis^∍^** | |  |  |
|  |  | **NO**  **N=56** | **YES**  **N=44** | **p-value** |  |
| Rehospitalization according to the VARC-3 criteria, n (%): | 10 (10) | 6 (10.7) | 4 (9) | 0.99 |  |
| - Procedure-related or valve-related hospitalization | 6 (6) | 4 (7.1) | 2 (4.5) | 0.92 |  |
| - Other cardiovascular hospitalization | 4 (4) | 3 (5.3) | 1 (2.2) | 0.81 |  |
| Stroke, n (%) | 0 (0) |  |  |  |  |
| TIA, n (%) | 5 (5) | 3 (5.3) | 2 (4.5) | 0.68 |  |
| NYHA III/IV, n (%) | 4 (4) | 4 (7.1) | 0 (0) | 0.21 |  |
| Bleeding events n, (%)^*^ |  |  |  |  |  |
| Type 1 | 2 (2) | 2 (3.5) | 0 | 0.60 |  |
| Type 2 | 0 (0) |  |  |  |  |
| Type 3 | 0 (0) |  |  |  |  |
| Type 4 | 0 (0) |  |  |  |  |
| New-onset atrial fibrillation, n (%) | 2 (2) | 1 (1.7) | 1 (2.2) | 0.90 |  |
| New-permanent pacemaker, n (%) | 24 (24) | 14 (25) | 10 (22.7) | 0.99 |  |
| Values are reported as number and percentage (%).  ^*^Defined according to the Valve Academic Research Consortium-3 definition. | | | | | |

**SUPPLEMENTARY FIGURE**

**Supplementary Figure 1. Commissural Alignment.**

Example of severe commissural misalignment.

**Supplementary Figure 2. Leaflet Expansion.**

*Asymmetrical leaflet expansion* was calculated by evaluating the angle formed by the border stent struts relative to each leaflet and the transcatheter aortic valve center point, represented by the coaptation level. Full leaflet expansion would be assumed to be 120° and asymmetric leaflet expansion is calculated as the sum of the difference between 120° and each measured leaflet angle.

Leaflets expansion was calculated as the sum of the difference between 120° and each measured leaflets angle: (120-126.7)+(120-114.7)+(120-118.7)=13.3

**Supplementary Figure 3. Implantation Depth.**

It was calculated form the pre and post multi detector computed tomography scan as the difference between the distance from the sinotubular junction (STJ) and the virtual basal ring (VBR) at the nadir of the left, right and non-coronary sinus and the distance from the STJ and the frame-inflow at the same nadir.

| **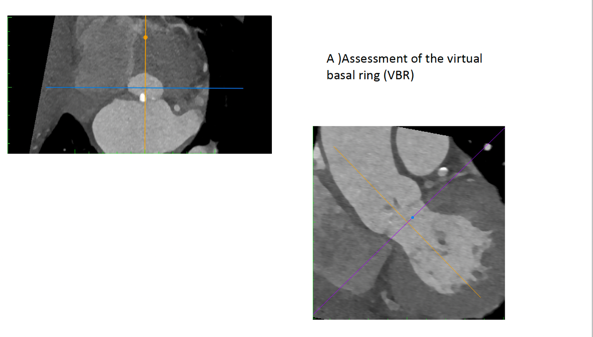** |
| --- |
| **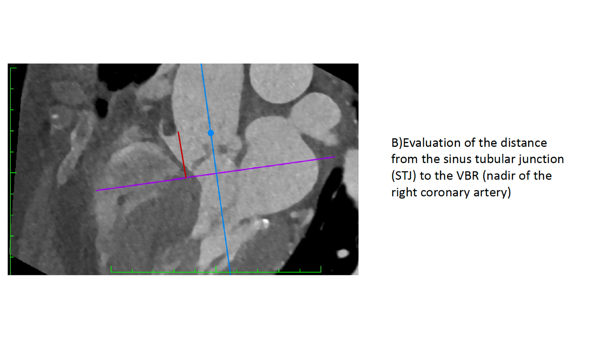** |
| **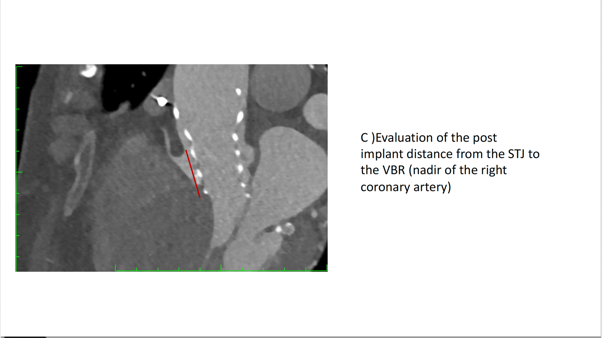** |
| **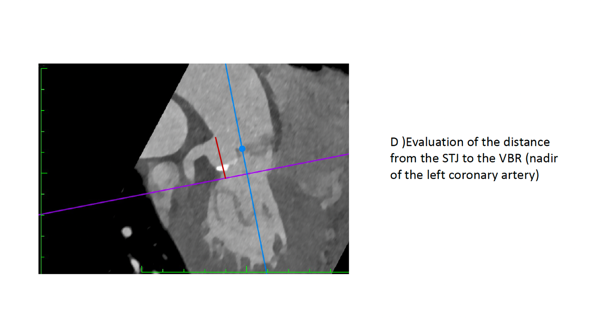** |
| **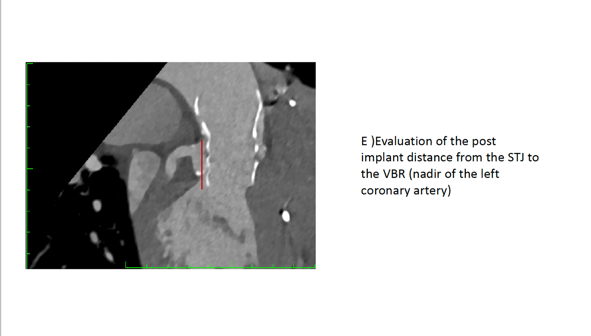** |
| **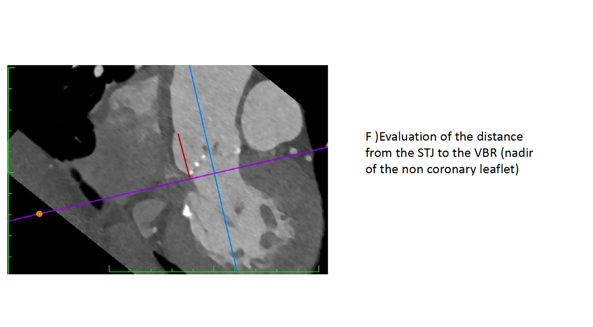** |
| **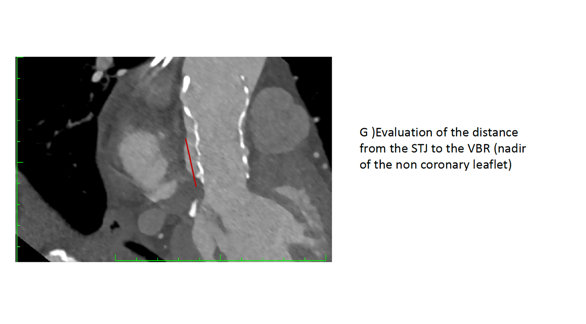** |

**Supplementary Figure 4. Valve to coronary and to non-coronary sinus distance.**

**A)**

**
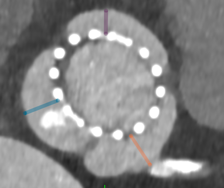
**

The left, right, and non-coronary *anatomic-sinus widths* were defined as the greatest distance measured from the prosthesis cage to the edge of the right, left, and non-coronary sinus, respectively.

**B)**

Valve to coronary and non-coronary distance was considered as a surrogate measure of the anatomic sinus, the space between the prosthesis cage/divider and the aortic wall.

**SUBGROUP ANALYSYS: HALT/SLT vs. rest of the population**

**Supplementary Figure 5: Mean gradient at follow-up HALT/SLT cohort vs. rest of the population.**

There was no difference in terms of mean gradient at six month follow up between HALT/SLT group (n=18) and rest of the population (n=82), 7.6$\pm$5.2 mmHg vs. 7$\pm$3 mmHg, p=0.64.

**Supplementary Figure 6: Effective orifice area (EOA) at follow-up HALT/SLT cohort vs. rest of the population.**

There was no difference in terms of EOA at six month follow up between HALT/SLT group (n=18) and rest of the population (n=82), 1.88$\pm$0.29 cm^2^ vs. 1.86$\pm$0.40 cm^2^, p=0.84.

**Bioprosthetic valve disfunction (BVD) incidence between HALT/SLT vs. rest of the population:**

|  | | | | |
| --- | --- | --- | --- | --- |
| **BVD** | **Overall**  **N=100** | **No HALT/SLT**  **N=82** | **HALT/SLT**  **N=18** | **p** |
|  | n=17 (17% | n=15 (18.3%) | n= 2 ( 11.1%) | 0.698 |

**Survival analysis**

**Supplementary Figure 7: time to event analysis**

Time to event (neurological event (TIA/Stroke) and re-hospitalization)) analysis, stratified by the presence/absence of thrombus at any level.

Hazard ratio:0.86, 95%CI: 0.24-3.06, log-rank test p= 0.82.
